# Immersive virtual-reality computer-assembly serious game to enhance autonomous learning

**DOI:** 10.1007/s10055-021-00607-1

**Published:** 2021-12-23

**Authors:** David Checa, Ines Miguel-Alonso, Andres Bustillo

**Affiliations:** Departamento de Ingeniería Informática, Universidad de Burgos, Burgos, Spain

**Keywords:** Virtual reality, Educational game, e-Learning, Active learning, Computer science, Game engine, Head mounted display

## Abstract

Immersive virtual reality (VR) environments create a very strong sense of presence and immersion. Nowadays, especially when student isolation and online autonomous learning is required, such sensations can provide higher satisfaction and learning rates than conventional teaching. However, up until the present, learning outcomes with VR tools have yet to prove their advantageous aspects over conventional teaching. The project presents a VR serious game for teaching concepts associated with computer hardware assembly. These concepts are often included in any undergraduate’s introduction to Computer Science. The learning outcomes are evaluated using a pre-test of previous knowledge, a satisfaction/usability test, and a post-test on knowledge acquisition, structured with questions on different knowledge areas. The results of the VR serious game are compared with another two learning methodologies adapted to online learning: (1) an online conventional lecture; and (2) playing the same serious game on a desktop PC. An extensive sample of students (*n* = 77) was formed for this purpose. The results showed the strong potential of VR serious games to improve student well-being during spells of confinement, due to higher learning satisfaction. Besides, ease of usability and the use of in-game tutorials are directly related with game-user satisfaction and performance. The main novelty of this research is related to academic performance. Although a very limited effect was noted for learning theoretical knowledge with the VR application in comparison with the other methodologies, this effect was significantly improved through visual knowledge, understanding and making connections between different concepts. It can therefore be concluded that the proposed VR serious game has the potential to increase student learning and therefore student satisfaction, by imparting a deeper understanding of the subject matter to students.

**Supplementary Information:**

The online version contains supplementary material available at 10.1007/s10055-021-00607-1.

## Introduction

Virtual reality (VR) technologies used within different fields have recently been successfully integrated into education, teaching and training. While VR is not new, recent advances in its technology have improved interaction and lowered costs, making it increasingly attractive to scholars. The new standalone generation of VR Head Mounted Displays (HMDs) dispenses with the inconvenience of cables that limit freedom of movement. On the one hand, almost all published studies of VR applications and educational goals report a clear increase in user satisfaction compared with conventional teaching methodologies. But on the other hand, those studies fail to prove a measurable increase in learning rates when using VR applications (Checa and Bustillo 2020a). A tangible advantage in favour of immersive virtual reality as a reliable pedagogical tool over less interactive and conventional teaching approaches must be shown in terms of learning performance, in order to achieve universal recognition for immersive VR.

In the general context of the COVID-19 pandemic, undergraduate students have had little choice other than to face high levels of confinement and have seen their social life curtailed, including social interaction during their learning process. Online learning has become a major tool in their daily life, drastically reducing one of the main positive outcomes of the learning process: the emotional component of learning. Clearly, new technologies cannot provide this emotional component; however, VR might reduce its impact in student demotivation by means of increasing student satisfaction with the learning experience. The immersive sensations and feeling of presence associated with interactive VR experiences (Bhattacharjee et al. 2018) are a useful means of mitigating student isolation and the negative effects of demotivation. VR supported lectures open new opportunities of learning by doing, countering those negative effects, motivating students through practice-oriented learning content, often a preference among students.

When selecting the teaching goal for this research—computer hardware and its assembly—the central issue was student demotivation associated with non-practical contents. Computer hardware is a compulsory topic in many computer science degrees that is often presented to students from a very theoretical perspective, due to budgetary limitations. In this approach, student interaction with computer components is curtailed. Computer hardware assembly is one of the first topics to be taught in any introduction to computer science, so student interest in the topic can often be weaker, as they wish to move on to other more practical aspects. This loss of interest will affect the rate at which they learn other concepts that will be presented later on, such as programming and network security.

In this research, three different teaching methodologies are compared: a conventional teaching method, a serious game for desktop PC, and an immersive VR serious game. All of them were adapted both for confined and non-confined students. Therefore, its conclusions will be useful both for COVID-19 conditions and for standard life, considering that future educational standards will always insist on a major proportion of online learning time for undergraduate studies, in all likelihood higher than before 2019. Besides, special effort has been made to increase the sample size, so as to search for statistically significant differences between student learning rates depending on the teaching methodology. Sample size is one of the main differences with previous works, where small samples of between 16 (Zhou et al. 2018) and 27 (Ajay et al. 2015) were selected for the experimental group. Therefore, this sample size of VR participants (*n* = 40) is to the best of the authors' knowledge one of the most extensive for learning tasks in a homogenous population (Checa and Bustillo 2020a).

Finally, the development of immersive VR serious games is still neither an easy nor a straightforward task. Usually, game developers and teachers who use games in their lectures are not within the same work teams. Therefore, teachers are limited to the use of existing games (Jensen and Konradsen 2018), limiting their capabilities of optimizing the learning experience. The skillset needed to develop VR environments is still very limited, despite the release of affordable VR creation suites. In view of these limitations, the two objectives of this research are to develop an immersive VR serious game associated with computer hardware concepts that can accentuate learning outcomes and to make it accessible to undergraduate students. At the same time, the entire virtual environment and their interfaces were also adapted to a second version of the game for desktop PCs. The game comprised three stages: (1) a tutorial helps the student to get used to the VR interfaces; (2) a second tutorial helps the student to learn the main concepts of computer hardware assembly where some predefined steps must be followed; and (3) the student completes the autonomous assembly of a computer with some pre-defined features, where the student has full freedom to interact with different hardware in a virtual lab.

The objectives and novelty of this research refer to VR learning outcomes, while keeping in mind the role that the serious game design plays in these outcomes. Up until the present, the learning capabilities of VR serious games have mainly been harnessed for training students to accomplish tasks; final evaluations involving repetition of those tasks. In this research, however, the academic accomplishments of the students will involve recalling outcomes, understanding skills and visual recognition of components not presented in the same form during the VR experience. In this way, our research is focused on the capabilities of VR serious games to generate new knowledge for the student. The higher cognitive load of VR learning experiences compared with 2D experiences and conventional teaching methodologies may be expected to enable a deeper understanding of the subject matter under study. The added-value of a comparison between 3 different learning methodologies in an extensive group of students is to assure the significance of the extracted conclusions. At the same time, the importance of a properly designed VR serious game is pondered in this research, so that high levels of student satisfaction and game usability are assured, as well as gaming applications not only in VR environments, but also in 2D screens for a broader use, especially in case of student confinement.

The remaining sections of this paper will be organized as follows: in Sect. 2, the most recent works on immersive virtual-reality serious games to enhance autonomous learning will be presented. In Sect. 3, the development of the immersive VR serious game for computer hardware learning will be described. In Sect. 4, the evaluation process and the learning experience performance will be described, after which the results of the learning experiences will be presented in Sect. 5. In Sect. 6, a detailed comparison will be presented between these results and recent related works. Finally, the conclusions and future lines of work will be presented in Sect. 7.

## Related work

The teaching goal of identifying and assembling computer hardware components that is proposed in this research could be considered as a mere assembly task. Some previous studies have proposed VR serious games as suitable tools for learning by building devices. Ajay Karthic et al. (2015) focused their research on teaching procedural skills and information. They compared VR devices with desktop solutions for learning how to perform a product functional analysis; the analysis task consisted of assembling the components of a coffee maker. Their study concluded that the performance outcomes (assembly time) using immersive VR systems was significantly better than using non-immersive VR systems. This result cannot be directly extended to other VR experiences, because the study had some limitations: it used a standard joystick interface (a low usability device compared with new interfaces) and the 54 students showed a broad age range (10 years) and a strong gender imbalance (13% female). Zhou et al. (2018) used an educational computer-assembly application to explore the influence of virtual reality on user game experience. They concluded that the use of VR heightened learning interest and fostered engagement, although any analysis of learning rates associated with different teaching methodologies was not approached. Finally, Zhou et al. (2018) concluded that students using VR took the same time to perform an assembly task as other students who had no previous practice with real components, while Koumaditis et al. (2020) reached the opposite conclusion. They concluded that study groups of twice the size (33 users compared with 16 people in the case of Zhou et al.) performed complex mechanical tasks (*e*.*g*., assembly of a 3D cube) in reality with greater efficiency in statistical terms than in VR environments. Therefore, many questions remain open concerning the effect of VR educational applications on learning rates and the influence of VR interface usability on learning outcomes, which may be partially resolved with larger sample sizes.

Computer hardware, it may be remarked, might not be the best topic to teach through immersive VR learning experiences. Any other topic with a closer relation with spatial elements may be more suitable and provide better learning scores when using VR, due to higher spatial visualization in VR environments (Molina-Carmona et al. 2018). Most of these topics may belong to Medicine (Moro et al. 2017), Mechanical Engineering (Wolfartsberger 2019), Architecture (Kowalski et al. 2020) and Cultural Heritage (Checa and Bustillo 2020b). However, the use of VR tools for computer science learning (Pirker et al. 2020) is not new in itself. Most of these studies identify important advantages when VR devices are used. Akbulut et al. (2018) found students who had a VR experience based on the concepts of software engineering scored higher than students who did not undergo VR learning. The use of analogies and metaphors to build mental models can benefit from the use of virtual reality, as experiences that teach theoretical concepts have shown such as finite state machines and object-oriented programming (Dengel 2019; Tanielu et al. 2019). The findings of other research (Greenwald et al. 2018), which compared VR fundaments of science learning with desktop-based VR and 2D images, showed no clear advantage of VR-based instruction. Considering Bloom taxonomy (Bloom 1956), some of these studies reported basic learning objectives, like remember, to describe educational goals; for instance, remember firewall filtering rules (Puttawong et al. 2017). Other studies focus on understanding concepts, such as finite state machines (Dengel 2019) or fundamental programming principles (Horst et al. 2019). Finally, some approaches focus on higher cognitive levels, such as creation in the sense of inventiveness (Bujdoso et al. 2017). As a further step in researching, this work tackles not only one of these categories but a combination of them, in order to achieve further educational goals. Secondly, former works focus on student´s capability to remember specific tasks, previously trained in VR environment. Contrarily, this research targets the potential of serious VR games to help students acquire new knowledge not directly provided in the same form; in other words, the student´s skill to generalise knowledge from specific examples. Therefore, visual recognition of a limited set of physical elements plays a secondary role in this research as opposed to other literature, in which the success of learning outcomes is assessed in terms of tasks trained by using the same set of physical elements. Finally, previous works did not compare the learning outcomes provided by different teaching methodologies. In most of them, VR is the only tested teaching methodology (Bujdoso et al. 2017; Dengel 2019; Puttawong et al. 2017). Only in a few cases, like (Horst et al. 2019), VR is compared with other methods, like desktop serious games, but traditional learning methodologies are never used for baseline comparison. To overcome this limitation, this research tests three different teaching methodologies to identify their advantages and drawbacks when acquiring different types of knowledge.

## Development of an immersive virtual reality serious game

The VR-serious game used in this research was designed following a previously presented design methodology (Checa and Bustillo 2020a). This methodology is composed of three stages: pre-design, game development and game evaluation. In the pre-design, a clear and testable learning hypothesis or objective is defined. The hypothesis is based on mainstream modern learning theories. During the game development, game and instructional features are developed while programming the serious game in the game engine. Game features should promote motivation to learn, while minimizing the entertainment impact (extraneous processing). Instructional features should increase the instructional impact (essential processing) without reducing the motivation (generative processing) as Mayer and Johnson stated (Mayer and Johnson 2010). Finally, student performance with the serious game is evaluated in a third stage, by measuring whether there has been an improvement in the learning outcomes. A learning outcome is a change in knowledge caused by instruction. The evaluation is divided into two issues: (1) measurement of student satisfaction; and (2) measurement of learning outcomes. Student satisfaction includes the evaluation of satisfaction, usability and simulation sickness, which was assessed at the end of VR experience.

### Pre-design

Following this methodology, the first step was to develop a clear and testable hypothesis. The subject "Introduction to Computer Science" is a mandatory study unit on all the engineering degrees and others, such as the Degree in Media Communication, taught at the University of Burgos (Spain). In addition, this subject is particularly complex to overcome for many students, due to the diversity and abstract nature of its contents. One of the first learning topics in this subject is computer hardware assembly, a topic that includes knowledge of computer components and their functionality, performance units, range of variation depending on the final use of the computer, etc. These concepts are currently supported by presentations that include computer images, data tables and diagrams. Unfortunately, practical exercises with these contents might require sufficient computers for each student to have one to disassemble, to extract components and to replace them, which are not always available due to cost constraints. In this research, the aim is to create an educational game that prompts students to interact with these concepts, so that they are learnt in a practical way. Moreover, the game should be an attractive and dynamic tool for students, that introduces them to the subject with positive learning results, providing additional motivation to work towards better grades on the course.

Three well-defined learning theories and one custom-designed model were followed to design the serious game as a suitable educational resource. Learning theories can be defined as proposals related to the way students assimilate, process and retain the information they have learnt, and provide guidelines on students' motivations, learning process and learning outcomes (Pritchard 2017). First, the theory of Liu et al. (2017) identified constructivism, autonomous learning, and cognitive load theory as the most suitable issues for VR serious games. Secondly, the technological perspective of the 3D Virtual Learning Environments (Dalgarno and Lee 2010) was also taken into account. This theory focuses on representational and interactive fidelity; the learning benefits in this theory are split into: representations of spatial knowledge, experiential learning, engagement, contextual learning and collaborative learning. Thirdly, Dale's Cone theory (Dale 1946) can also be suitable for VR serious games. According to this theory, students learn best when they go through a real experience or the experience is realistically simulated. Finally, our research follows the operational learning model proposed by Zhou et al. (2018) that proposes a learning model in which aspects of human–computer interaction and pedagogical aspects are merged, considering logical contexts, roles and scenarios of VR environments. This model uses constructivism at the abstract level, because the contexts, activities and social interactions in the learning environment promote the construction of new knowledge. Students therefore learn through autonomous interaction, hands-on learning, and problem solving. VR technologies offer (1) realistic experiences in which to practice these principles, (2) a safe environment where mistakes can be corrected, and (3) immediate feedback provided on operational learning.

### Game development

**Fig. 1:**
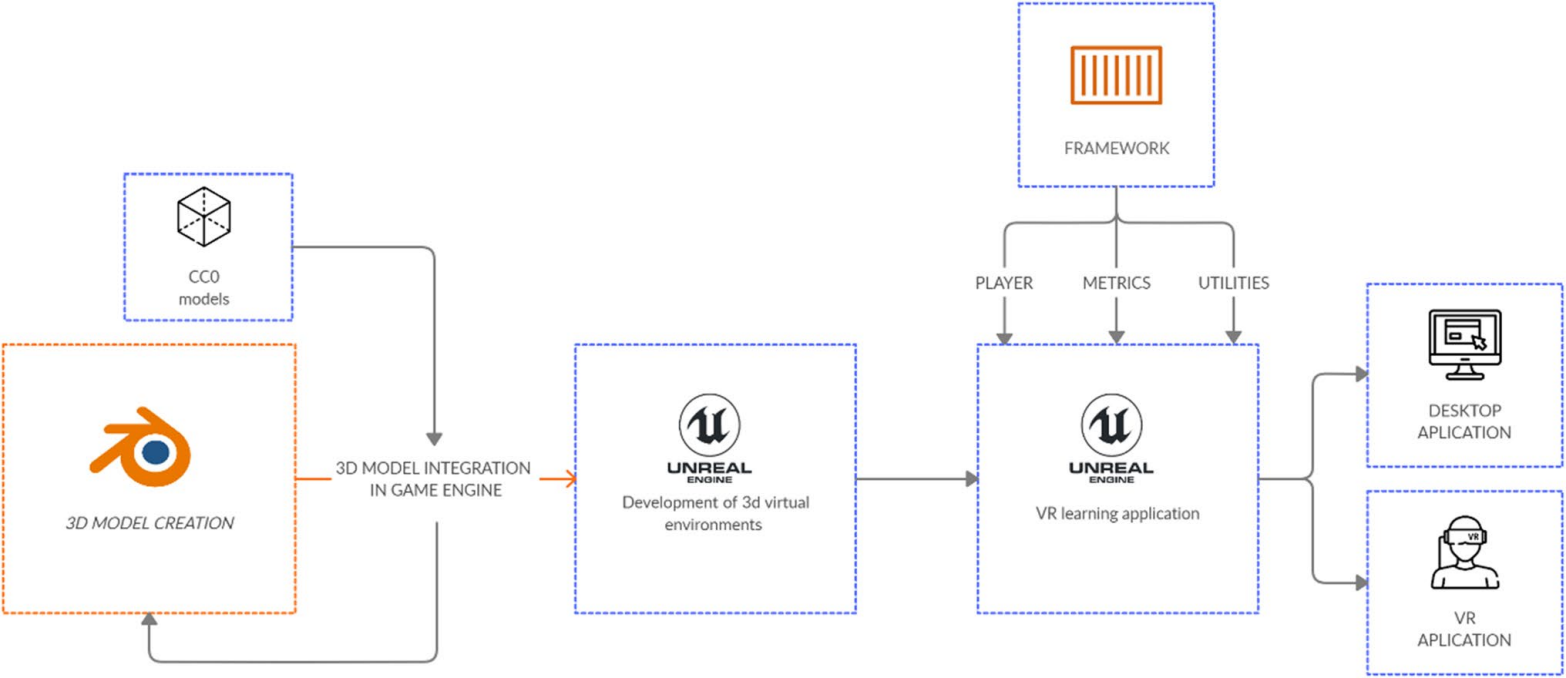
Pipeline of game development

Roussos et al. (1999) defined four key objectives for the design of a VR serious game: interaction, immersion, user participation and, to a lesser extent, photorealism. The accomplishment of all these objectives is possible with new game engines and HMD devices. They present very high resolution, wider field of view, ultra-precise tracking and 6 Degrees of Freedom (DoF) interaction elements, creating a very strong sense of presence and immersion. The steps followed to develop the VR serious game are summarized in Fig. 1. They include: (1) 3D model creation, (2) integration of these models in the game engine, (3) development of the 3D virtual environments, (4) creation of the VR learning experience, and (5) adaptations for VR and desktop applications.

**Fig. 2:**
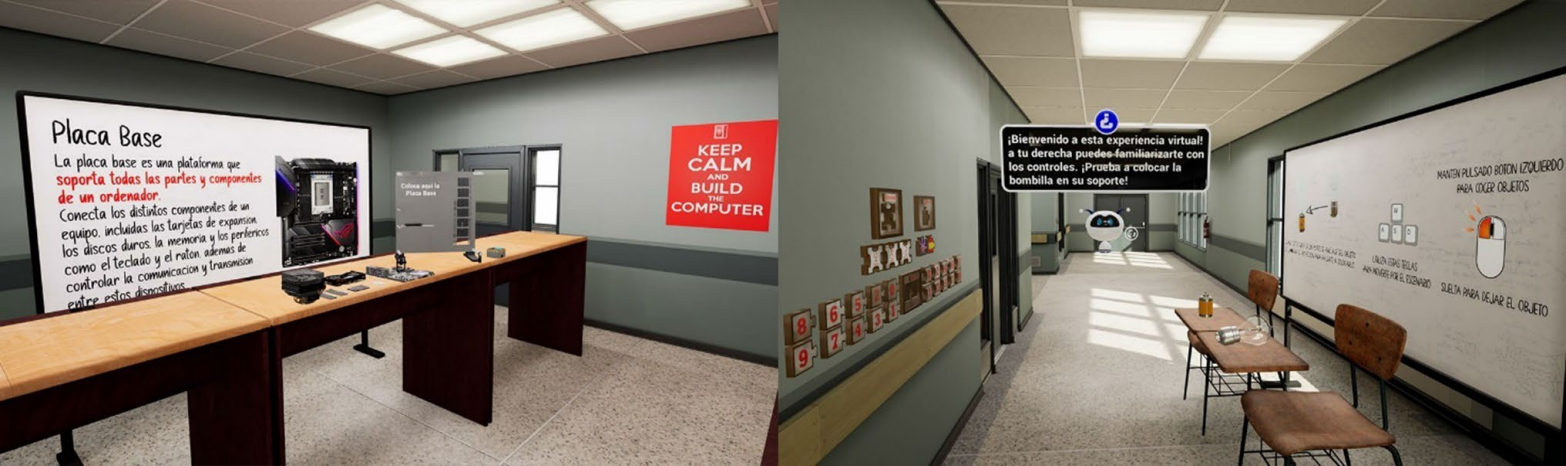
Examples of the 3D models and the virtual environments included in the serious game

Blender software was applied to create the 3D models. Besides, some 3D models, released under CCO licenses, were also downloaded from different sources. These models were integrated in the game engine. Unreal Engine™ was chosen, due to its high capacity to create photorealistic environments and its visual scripting system, blueprints that are used to create very complex experiences with little or no knowledge of programming languages, as former works have demonstrated (Checa et al. 2020; Checa and Bustillo 2019). 32 unique PC hardware objects were modelled and categorized in 8 different sections: CPU, CPU Cooler, GPU, Hard Drives, Mainboards, RAM, power supply, and PC Cages. Only one classroom and one corridor were modelled for the environment. Figure 2 shows some examples of the 3D models and the virtual environment.

When creating VR learning applications, the first step is to choose the type of VR experience properly. VR learning applications can be divided in four types: Explorative Interaction, Explorative, Interactive Experience, and Passive Experience. In Explorative Interactions, the user explores the environment and interacts with it freely. In contrast, an explorative experience simply involves free exploration, although without direct interaction. The Interactive Experiences permit user interaction, but no free movement. Finally, the Passive Experience is the most limited one, in which user interactivity and movement are very constrained. Most VR learning applications use passive experiences, because they are more easily created with low-cost solutions. However, although student satisfaction is improved, significant learning improvements are not achieved (Checa and Bustillo 2020a). Therefore, Interactive VR Experiences are currently the most appropriate type, because they show a good balance between cost, current technology development, immersion, and interactivity. This solution is more cost-effective than Explorative Experiences and is therefore the type of serious game selected in this research.

The VR serious game was developed with the support of a previously created framework (Checa et al. 2020). The framework simplifies the game development process with functions and services that are pre-programmed for their effective reuse such as player utilities, an evaluation manager, and tools for metrics. This framework also makes it possible to have a single project that can be played on a 2D screen or on VR devices, merely by changing the pawn that automatically detects whether or not the user has connected a compatible VR device. On the one hand, if the user plays on a 2D screen, the interface will be the mouse and the keyboard; on the other hand, if a VR device is used, the interaction will be through hand controllers. The game works with Oculus Touch controllers, used in this study, but it is also compatible with HTC Vive and Windows Mixed Reality headsets and controllers. Game design decisions were oriented to achieve natural interactions as well as to mitigate any usability constraints of the controllers. The game design was focused on usability, with a limited interaction technique in VR, to accelerate the learning curve of the user: among the 3 basic forms of interaction techniques in VR (selection, manipulation and locomotion), the user only had to focus on the manipulation of objects. A single mechanism was used to pick up and to drop objects as well as to place them in position. The user had to press and to hold the controller trigger to pick up objects and the hand dropped the objects when the button was released. An attachment system was programmed to help the user to place the object in the desired position when it was sufficiently close to the attachment point, to facilitate the assembly task of the computer. The outline of the hand was also highlighted in a light green colour whenever within range of an object, to facilitate grabbing. The objects floated back to their original locations a few seconds after being released, to prevent users from accidentally dropping or throwing parts away. This design solution avoided the use of a specific button-based mechanism that could be difficult for a novice user. Finally, a first level ensured that the implementation of the interaction could not interfere with its performance where users might become accustomed to this method of interaction.

**Fig. 3:**
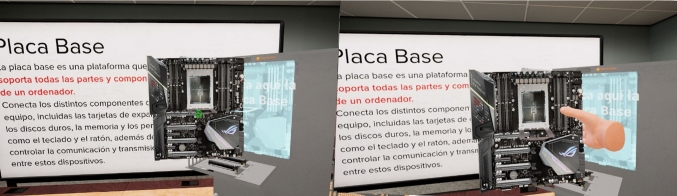
User interaction with the mouse and keyboard in the desktop game (left); controller interaction in the VR game (right)

If played on 2D screens, the interaction in the game is controlled with a keyboard and a mouse, using the left click to pick up, to drop and to place objects. The movements use the arrows or AWSD of the keyboard. Figure 3 shows a user interacting with the game with a VR device and the same action in the desktop version.

**Fig. 4:**
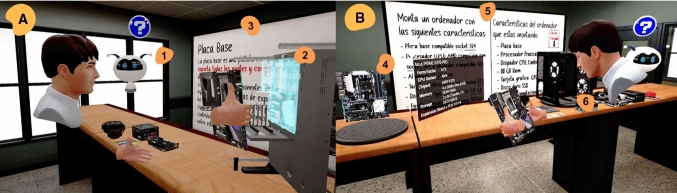
**A**: tutorial level (left) A1: assistant robot, A2: test bench where the user places the different computer elements, A3: Board showing the main information of the grabbed component. **B**: assignment level (right). B4: information location that shows relevant characteristics of the hardware, B5: board with the requested requirements, B6: variety of hardware and cage in which to place it

As Fig. 4 shows, the VR experience was designed so that students could progress through different levels and advance towards the following goals:

- Introduction: At this level, the user follows a semi-guided tutorial to learn how to use the VR interface as well as the mechanics, such as grabbing and placing objects, that will be used in the game later on. The novelty of the VR environments and interfaces can limit the user's learning experience, especially if they have never used those devices before or are unfamiliar with them. Known as the "novelty effect" or Hawthorne effect, this issue refers to the way the virtual information on display and the technology may distract students (Looi et al. 2009).The introduction that has no time limit is an attempt to circumvent the novelty effect, giving the user sufficient time to become familiar with the technology and interfaces.
- Tutorial: the student, guided by the virtual instructor, has the task of assembling a computer. The tutorial is a fixed step-by-step process where students receive continuous feedback and help from the assistant robot (spot 1 in Fig. 4A) to learn where each component should be placed. This assistant only displays information when the student looks directly at it, helping to avoid visual overload of the space, which could adversely affect student attention levels. The student has to slot the different computer components into place according to the design (spot 2 in Fig. 4A). The board, situated in front of the assembly table, serves as a reminder of the academic content, showing the main information on the component that the student is positioning in real time (spot 3 in Fig. 4A). This step also serves to circumvent the novelty effect and initial astonishment at the VR environment that can reduce the attention levels of students when focusing on the learning experience in the next step. It also helps students to become familiar with the information and the way it is presented, thereby mitigating, in the final assignment, the distraction of the virtual information on display.
- Assignment: through self-instruction, the student has the task of assembling a computer, which involves the selection of mutually compatible components, their proper positioning, and their connection in a reasonable order. There is a wide variety of hardware from which to choose, although the PC to be built must meet certain requirements (spot 5 Fig. 4B). The student can check the specifications of each component, in order to perform this task successfully, placing the component in an information location on the assembly table (spot 4 in Fig. 4B). In this way, the student will be able to choose the right component and can proceed to assemble the hardware in the cage (spot 6 in Fig. 4B). The required specifications of the computer are shown to the left of the whiteboard, while on the right-hand side, the user can see the specifications of the computer once it has been assembled and can check whether the right components have been used.

This experience has been created for both desktop (2D screens) and immersive virtual reality versions. The desktop version works with any computer with medium processing requirements and has been optimized to run at over 90 fps on an Intel^R^ Core™ i5-8600 processor, minimum 8 GB RAM and NVIDIA GTX 1050. Immersive virtual reality version has more hardware requirements and has been optimized to work properly on computers with at least 16 GB RAM and NVIDIA GTX 1070 graphic cards. Both versions of the serious game can be downloaded from the following URL https://3dubu.es/en/virtual-reality-computer-assembly-serious-game/. Besides, this webpage includes several videos of students playing the serious game in virtual reality and on desktop PCs that provide a broader vision of the performance of the game.

Finally, as the game experience was to be compared with a conventional lecture, a lecture experience was also designed. In this lecture, the teacher presents the computer hardware components in detail, using an open computer to interact with the hardware, while the student is unable to manipulate the components. In this lecture experience, the teacher followed the same steps for assembly as were in the game, considering hardware limitations (no extraction of screwed components). The students’ role was limited to viewing the teacher’s actions, through a webcam broadcast, and listening to the explanations. Although the students were sitting in the classroom and a beamer was projecting the webcam broadcast, the broadcast could just as easily have been followed through web services in case of student confinement. The event was baptized the webcam experience, as a webcam was used to display teacher interaction with the computer.

## Learning experiences and evaluation process

Following Mayer’s proposal of teaching experience design (Mayer 2014), any teaching experience should be compared with valid, reliable, objective and referenced instruments with experimental control groups that use conventional modes of teaching. In this research, the VR serious game was compared with two conventional teaching methodologies. First, the same game was used with one control group but playing in desktop PCs. This group will benefit from some of the advantages of a serious game: autonomous learning, hands-on motivation and problem solving. However, this group also has some limitations: the mouse and the keyboard are used as the game interface, which is a slightly less natural interface, and users miss the benefits of immersion and presence associated with VR environments. The second control group enjoys a learning experience based on an instruction class that explains the main components inside a computer by displaying its different parts in great detail through a webcam that projects the images on a large screen. The computer has most of its components disassembled and during the class students watch as many components are assembled: the CPU dissipator, RAM, hard drives, power supply, and graphics card. The duration of this class was 25 min, which is very close to the average time that the other groups spent playing the game.

The study was conducted as part of the Introduction to Computer Science subject in the Media Communication degree of the University of Burgos. The study sample consisted of 77 first-year students (mean age = 18.6 years old, 37 male and 40 female). The participants were randomly assigned to three different groups: Virtual Reality group (40 students), Desktop PC group (19 students) and Webcam Experience group (18 students). All relevant dimensions of both the treatment and the control groups were equivalent (Mayer 2014), while the Virtual Reality group was larger, because is the one under study and a higher variability is expected. Although the two control groups (Desktop and Webcam groups) initially had 20 students, some students were omitted, because they had missed some of the lectures or they had not filled in the tests. The size of each group was defined by taking into account the following design factors: 1) number of learning methodologies to be compared; 2) the homogeneity of the groups of students; 3) higher expected performance variability in the VR experience, due to its novelty; 4) the statistical techniques (ANOVA and ANCOVA) for the results analysis, 5) the limited effect of the learning experiences on academic outputs, due to their short duration, and 6) a minimum of 15 students in the reference group. These criteria are properly derived from the existing bibliography (Birckhead et al. 2019; Gall et al. 2003). Then, the availability of students and their separation into practical groups were considered to fix the final size of each group for each learning methodology.

**Fig. 5:**
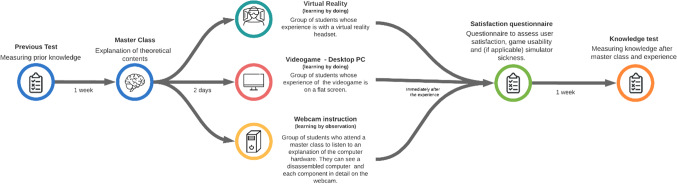
Design of the whole learning experience including evaluation stages

The learning experience includes four stages: previous test, master class, learning experience itself, satisfaction questionnaire and, finally, a knowledge test. Figure 5 summarizes the workflow of this process. All the students received identical treatment, except for the type of experience they performed (VR, desktop PC or webcam-based experience).

The learning experience began with a pre-test to assess the previous knowledge of students. It consisted of 9 Multiple Choice Questions (MCQ) and one image-based question to identify the different components of a computer viewed without one of its side panels. The pre-test took place before the students had received any lecture on computer hardware and can be consulted in the Appendix I. Although we can find conflicting positions on the advantages and disadvantages of using MCQ, in general, it has been considered more appropriate to test large amounts of surface knowledge throughout a study unit (Excell 2000). A week later, the theoretical contents that constituted this didactic unit on computer hardware were explained in the form of a master class with the learning objectives “Recalling” and “Understanding”. Two days later each group carried out the corresponding learning experience: playing the serious game in VR or the same game on a desktop PC or giving group presentations in class to explain the internal positions of computer components while observing webcam images of its parts. Immediately after the experience the students from the first two groups filled in a questionnaire to assess user satisfaction with the experience, game usability, and simulator sickness. The usability questionnaire was adapted from (Tcha-Tokey et al. 2016). The learning objectives that are associated with the serious game are “Understand” and “Create”. The surveys administered to each group can be consulted in Appendix II.

**Fig. 6:**
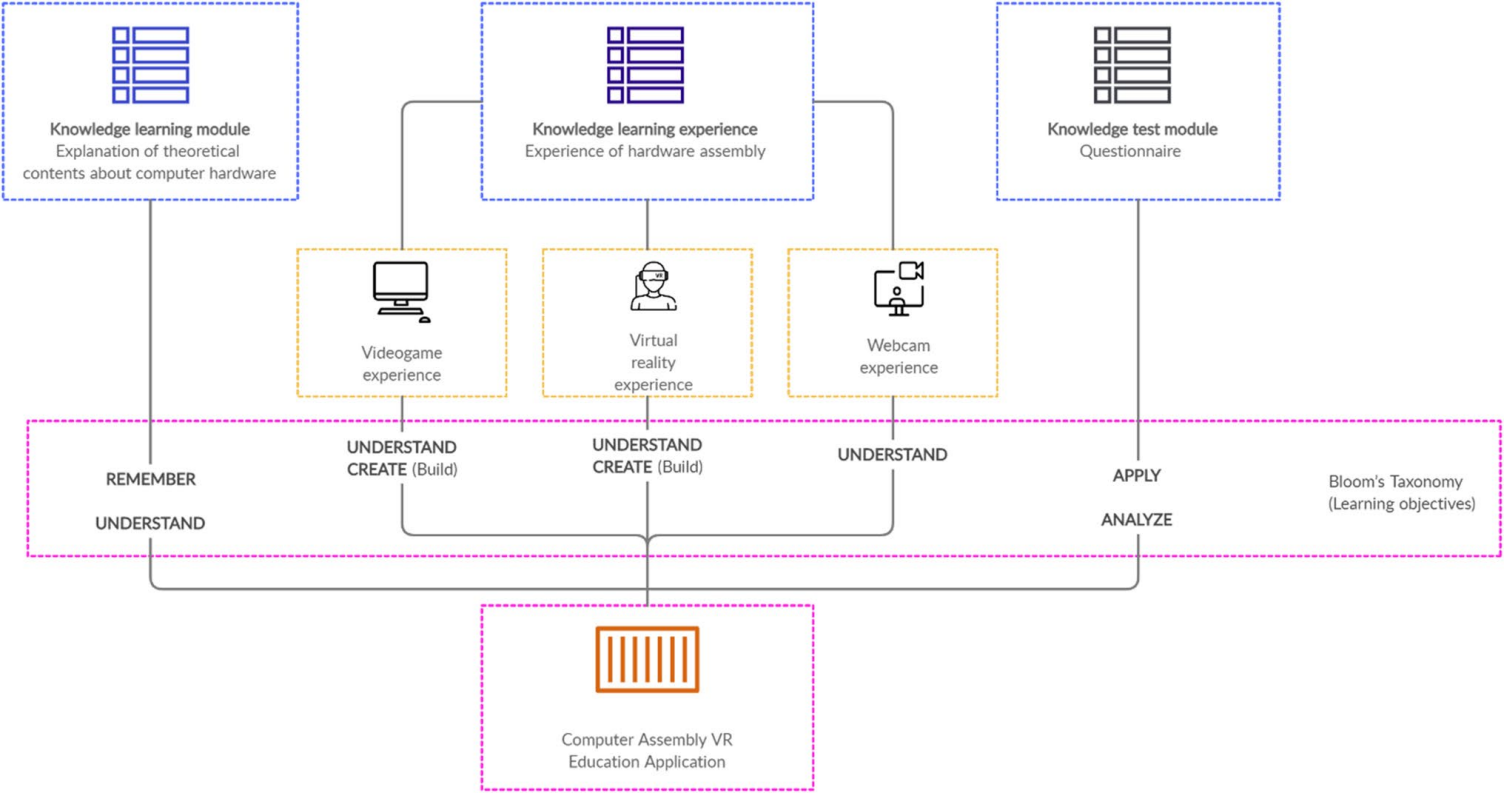
Operational learning model used based on the proposed learning model in Zhou et al. (2018)

Finally, a week after the experience, a knowledge test was performed. The test was not completed immediately after the experience, because delayed tests are particularly useful when determining the persistence of learning outcome effects (Mayer 2014). If administered immediately after the experience, much of the information may still be stored in the short-term memory, so the test results might not reflect comprehensive learning or long-term retention. The test included different questions from the pre-test, because equal tests can affect the learning evaluation. A pre-test can also serve as a learning episode (Johnson and Mayer^ 2009^), and if the same test is used as post-test, it can often lead to better marks than questions drawing from the learning experience. The post-test contains 21 questions: 15 multiple-choice type questions, 4 open questions, the answers to which are a chance to demonstrate conceptual knowledge through extended explanations and that probe student understanding of the educational content. Finally, the last 2 questions refer to PC images in which users have to write the name of each component in an empty blank. The test was designed to respond to a multi-level assessment by focusing on retention (recalling essential information), transfer (capability of using the learning information to solve new problems and to adapt to new situations) and understanding. The questions were divided into two categories on the basis of Bloom’s taxonomy (Bloom 1956). The first group (7 questions) were related to recalling or remembering information. The second group of 6 questions referred to the understanding of information, in particular from class discussions, and conceptual knowledge. In addition, the two image-based questions served to identify, locate and recognize different components of a computer. The higher learning objectives associated with the post-test were “Apply” and “Analyze” and they can be consulted in Appendix III. The operational learning model used in this research is summarized in Fig. 6.

**Fig. 7:**
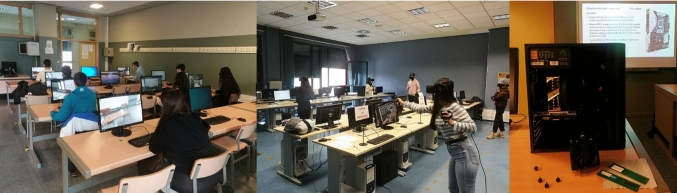
The three educational experiences

The procedure followed in the learning experiences changed with each group. The VR group used Oculus Rift HMD and Oculus Touch controllers. Five stations were set up with computers equipped with Intel^R^ Core™ i7-4790 CPU 3.60 GHz, 16 GB RAM, with NVIDIA GTX 1070 graphic cards. The procedure followed the recommendations for the prevention of COVID-19 transmission. Several consecutive days were necessary for these preventive measures, so that all students could take part in the experience. The Desktop group used the computers equipped with an Intel^R^ Core™ i5-8600 processor, 8 GB RAM and NVIDIA GTX 1050 available in the regular classroom. Finally, for the webcam experience group, we used the normal classroom with the help of the projector connected to a webcam. Figure 7 shows the 3 learning experiences as they took place.

## Results of the learning experience

In this section, the results obtained with the different evaluation tests and the other performance indicators will be collected. The raw data and their analysis presented in this section can be found in Appendix IV. The data were analyzed at *α* = 0.05 using the XLSTAT Statistical Software version 2018 (Addinsoft, New York, NY, USA) and are presented in terms of their Mean (M) values and Standard Deviation (SD). Welch's statistical tests were applied in the analysis to check for any possible sample imbalance.

### Satisfaction with the experience

All the groups were administered a short survey to measure student satisfaction with the educational experience, immediately after the experience. The first survey questions were on their satisfaction with the experience (0-very low to 5-very satisfied) and then on their learning belief concerning the learning experience and its suitability for acquiring the required knowledge (0-not useful to 5-very useful). A one-way ANOVA based on the survey results yielded the information summarized in Table 1. A significant difference between the satisfaction of the VR group (*p* < 0.0001) and the Desktop group (*p* = 0.003 < 0.05) compared with the Webcam group was found. These results indicated that the use of a serious game in the learning experience in both the desktop and the VR versions produced a significant improvement in student satisfaction compared to the traditional teaching method. The students also gave very positive learning belief ratings to the VR (*p* = 0.016 < 0.05) and the desktop versions (*p* = 0.036 < 0.05). The higher scores of the VR versus the desktop version of the game for both questions should also be highlighted.

**Table 1:** ANOVA of the satisfaction survey (M – mean value, SD—standard deviations) for VR, desktop, and webcam groups

Variable	Type of learning	N	M	SD	*p*
Satisfaction	VR	40	4.34	0.85	< 0.0001**
	Desktop	19	3.83	0.70	0.003*
	Webcam	18	2.94	0.96	
Learning beliefs	VR	40	4.56	0.70	0.016*
	Desktop	19	4.27	0.75	0.036*
	Webcam	18	4.05	0.65	

*p < 0.05, **p < 0.0001

### The usability of serious games

Four questions on the usability of serious games were included in the satisfaction questionnaire adapted from (Tcha-Tokey et al. 2016). All of them used a five-point Likert-type scale ranging (1-strongly disagree to 5-strongly agree). The first question asked users about how well they thought they controlled the game interface (Q3). The second asked whether the interactions seemed natural (Q6). The last 2 questions referred to the clarity of the goals and the physical configuration (Q4 and Q5). A one-way ANOVA was conducted, and the results, summarized in Table 2, showed that participants thought it was significantly more natural to interact in VR with Oculus Touch than on the desktop with the mouse and the keyboard. Previous investigations have arrived at similar results (De Paolis and De Luca 2020) that might explain the result of Q3, where the VR users were able to control the game slightly better than the desktop users. Finally, although the VR group understood the information displayed in the game, they expressed a more intense impression that they had been partially lost in at least one step of the game. These feelings may respond to a precarious game design at certain steps that should be remedied in future experiences.

**Table 2:** ANOVA of usability survey (M – mean value, SD – standard deviations) for VR, desktop, and webcam groups

Variable	Type of learning	N	M	SD	*p*
Q3: Have you been able to control the game without problems?	VR	40	3*.*48	0*.*86	0*.*215
	Desktop	19	3*.*16	0*.*98	
Q4: At each step, did you know what to do?	VR	40	3*.*29	1*.*14	0*.*773
	Desktop	19	3*.*38	1*.*24	
Q5: Is the information provided within the game clear?	VR	40	4*.*51	0*.*55	0*.*421
	Desktop	19	4*.*38	0*.*50	
Q6: Did the interaction with the virtual environment seem natural?	VR	40	4*.*24	0*.*79	0*.*015*
	Desktop	19	3*.*66	0*.*84	

*p < 0.05

### Performance in game

Some conclusions on user performance time at each stage of the game can be advanced. In Table 3, the ANOVA analysis is summarized. The time spent by the VR group was significantly longer at the introductory level. This level was used to enhance user familiarity with the environment and its physical configuration. In the second phase, a guided tutorial on PC assembly, the VR group was a bit faster than the Desktop group. Finally, in the Assignment phase, the VR group was significantly faster, finishing the proposed task more quickly. Besides, the game records the number of errors made in the Assignment phase. The VR group had an average of 2.1 errors per user while the Desktop group reached 2.8. So, the VR group was faster and more precise for computer assembly. A relationship can be established between the higher usability of the VR version with the Assignment time. In addition, although VR users were not familiar with the VR interface, they could clearly compensate that lack of confidence with an extra minute of training (Introduction level).

**Table 3:** ANOVA of performance times (M – mean value, SD – standard deviations) for VR and desktop groups

Variable	Type of learning	N	M (in minutes)	SD	*p*
Time in Introduction	VR	40	4*.*36	0.76	< 0*.*0001**
	Desktop	19	3*.*11	0.69	
Time in tutorial	VR	40	3*.*70	1.00	0*.*058
	Desktop	19	4*.*29	1.15	
Time in assignment	VR	40	12*.*95	4.73	0*.*017*
	Desktop	19	16*.*52	5.70	
Total time	VR	40	21*.*17	5.45	0*.*087
	Desktop	19	24*.*05	6.40	

*p < 0.05, **p < 0.0001

### Academic achievement

The pre-test included 10 questions that evaluated previous knowledge of computer hardware. A right answer was recorded as 1 and false ones as 0 and the responses to the 10 questions were averaged. Table 4 shows the average values and the one-way ANOVA. The low marks indicated that the students possessed insufficient computer-hardware-related knowledge before the learning experience, especially in the VR group. The non-significant effect between groups is required for a later comparison between group performance (Mayer 2014).

**Table 4:** ANOVA of the averaged pre-test questions for VR, desktop, and webcam groups

Variable	Type of learning	N	M	SD	*p*
Pre-test scores	VR	40	0.48	0.20	0.32
	Desktop	19	0.53	0.25	0.91
	Webcam	18	0.54	0.25	

The post-test included 21 questions. A right answer was recorded as 1, and if otherwise as 0. The analysis of the differences between the three groups was conducted with an Analysis of Covariance (ANCOVA) using the pre-test scores as the covariate and the post-test scores as dependent variables. Table 5 summarizes the ANCOVA results, in which the mean values of the post-test scores were 0.54, 0.50, and 0.47 for the VR, the Desktop and the Webcam group, respectively. Between the VR and Webcam groups a significant difference was found (*p* = 0.003 < 0.05), indicating that VR students showed significantly better academic performance than the group that received the traditional lecture. The Desktop group also showed slightly better marks than the Webcam group, but at some distance from the positive results of the VR group.

**Table 5:** ANCOVA of the post-test (M – mean value, SD – standard deviations) for VR, desktop, and webcam groups

Variable	Type of learning	N	M	SD	*p*
Post-test scores	VR	40	0.54	0.19	0.03*
	Desktop	19	0.50	0.17	0.62
	Webcam	18	0.47	0.19	

*p < 0.05

**Fig. 8:**
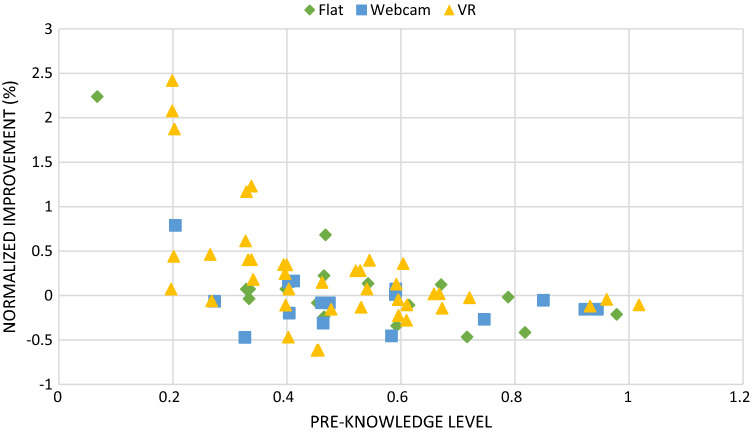
Normalized improvement in terms of participants prior knowledge

The graph plotted in Fig. 8 reflects an evaluation of this result, not only from averaged values but also from a general picture of each student’s performance. It shows (Y-axis) the improvement percentage for each student normalized to its averaged mark in the pre-test ((post-test − pre-test)/pre-test). On the X-axis, the pre-test average mark is plotted. An examination of Fig. 8 shows higher improvements (Y-values) in all ranges of pre-knowledge (X-axis) among the VR users. The students with very low marks in the pre-test especially showed higher improvements, which was a very interesting result that reflects the potential of VR for student learning of new though difficult hardware topics, opening up new avenues for future research with specific interventions with these sorts of students.

As stated before, the post-test questions were divided into different categories. The first group was related to recalling the information of the pre-test questions. The second group concerned the understanding of information and discussion. Finally, the third group was a set of image-based questions. A separate analysis was therefore performed for the 3 groups of questions, for an in-depth analysis of the results of the post-test.

#### Information recall

The questions forming this section (7 in the pre-test and 7 in the post-test) were focused on student ability to recall or remember essential information. The one-way ANOVA showed no significant effect between groups in the pre-test. An ANCOVA analysis was therefore performed using the pre-test scores as the covariate and the post-test scores as dependent variables, Table 6. Neither were significant differences found between groups. The Webcam group achieved the best averaged results, while the roughly equivalent results of the VR and the Desktop groups were slightly worse.

**Table 6:** Analysis of the answers of the VR, the desktop, and the webcam group to questions on information recall

Variable	Type of learning	N	M	SD	*p*
ANOVA analysis of answers to the questions on recalling information in the Pre-Test: Q2, Q3, Q4, Q5, Q6, Q7, Q8	VR	40	0.58	0.20	0.297
	Desktop	19	0.62	0.17	0.163
	Webcam	18	0.51	0.28	
ANCOVA analysis of answers to questions on recalling information in Post-Test: Q1, Q2, Q4, Q9, Q13, Q14, Q15	VR	40	0.52	0.20	0.304
	Desktop	19	0.51	0.26	0.201
	Webcam	18	0.55	0.26	

A final analysis of this group of questions was focused on the only two questions that were repeated between the pre-test and the post-test (Q3 and Q5). Those questions were the most complex, because less than half of the users answered them correctly in the pre-test (42% for Q3 and 41% for Q5). In both, 76% and 60% of the students answered those questions correctly in the post-test without any statistically significant difference between the three methodologies.

#### Understanding

This group of 6 questions was intended to measure the understanding of the information and, in particular, to give the possibility of discussing and demonstrating conceptual knowledge to the students. The one-way ANOVA showed an almost significant effect between groups in the post-test, between the VR and the Desktop groups and a slight better result than the Webcam group, Table 7.

**Table 7:** ANCOVA analysis of the answers of the VR, the desktop, and the webcam groups to questions on understanding in the post-test

Variable	Type of learning	N	M	SD	*p*
Answers to questions on understanding in the Post-Test questions Q6, Q8, Q10, Q17, Q18, Q19	VR	40	0.50	0.22	0.05*
	Webcam	18	0.42	0.19	0.64
	Desktop	19	0.37	0.23	

Within this category of questions, it is interesting to analyze the only question repeated from the pre-test: How long (in minutes) do you think it takes to mount two RAM modules and the graphics card in a computer? (Q17). The correct time was set at around 10 min. The one-way ANOVA, Table 8, showed no significant effect between groups in the pre-test. The ANCOVA analysis of covariance showed significant differences (*p* = 0.003 < 0.05) between the VR and the Webcam groups, highlighting the good performance of the VR group. Besides, the VR group was the one that came closest to 10 min and improved most with respect to the pre-test.

**Table 8:** Analysis of the answers of the VR, the desktop, and the webcam groups to Q17 from the post-test questions

Variable	Type of learning	N	M	SD	*p*
ANOVA analysis of the responses in the Pre-Test to the question on the time (in minutes) that it takes to mount the RAM and the graphics card of a computer	VR	40	22.8	19.25	0.20
	Desktop	19	13.17	10.82	0.49
	Webcam	18	16.68	17.11	
ANCOVA analysis of responses in the Post-Test to the question on the time (in minutes) that it takes to mount the RAM and the graphics card of a computer	VR	40	9.78	6.54	0.003*
	Desktop	19	14	12.18	0.170
	Webcam	18	17.35	10.91	

*p < 0.05

#### Visual recognition

**Fig. 9:**
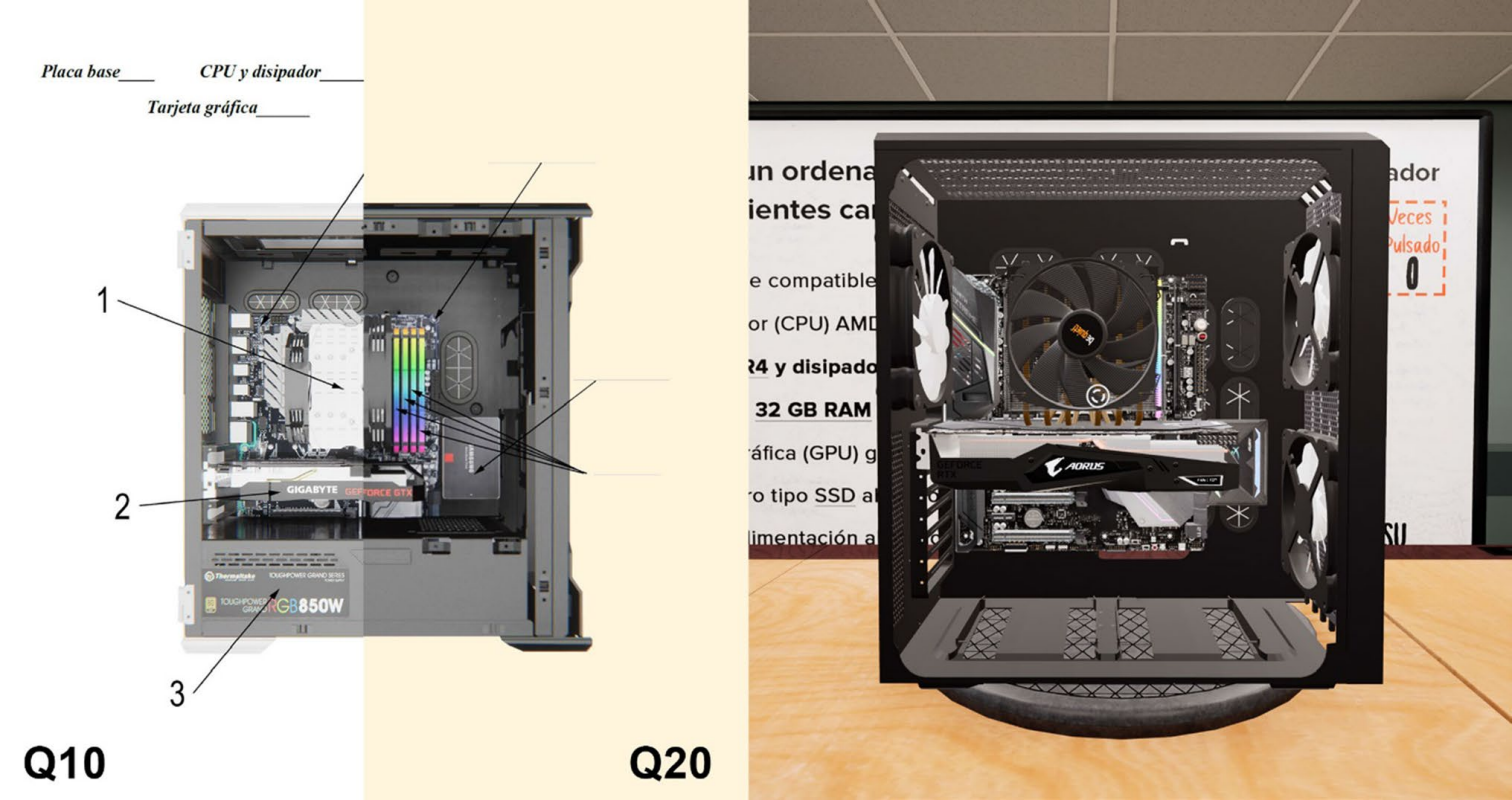
Q10 (pre-test) and Q20 (post-test (left) and computer cage in the serious game (right)

Finally, the category Visual recognition was based on assessing the capability of students to recognize and to locate computer hardware components. For this purpose, 6 questions were asked that addressed visual aspects of the hardware. Only one question was repeated between the two tests (Q10 in the pre-test and Q20 in the post-test), although its difficulty was slightly greater in the post-test. As Fig. 9 shows, in the pre-test, Q10 asks the user to select the right name of each component from a list, while in the post-test, Q20, the user only has a blank space on each component, and the proper name of the component should be recalled and written down. Besides, the computer images shown in the questionnaires are not exactly the same as the computer cage displayed in the serious game, as shown also in Fig. 9. The one-way ANOVA showed no significant effect between the groups in the pre-test. The ANCOVA analysis showed significant differences (*p* = 0.021 < 0.05), indicating that the VR group achieved significantly better visual recognition than the Webcam group, Table 9.

**Table 9:** Analysis of the answers of the VR, the desktop, and the webcam groups to the visual recognition questions

Variable	Type of learning	N	M	SD	*p*
ANOVA analysis of the answers to the Visual recognition questions in Pre-Test questions Q9, Q10	VR	40	0.39	0.26	0.17
	Desktop	19	0.39	0.28	0.23
	Webcam	18	0.50	0.31	
ANCOVA analysis of the Visual recognition questions in Post-Test questions Q5, Q7, Q11, Q12, Q20, Q21	VR	40	0.55	0.25	0.021*
	Desktop	19	0.53	0.28	0.098
	Webcam	18	0.44	0.27	

*p < 0.05

## Discussion

Firstly, the analysis of the results revealed significantly more satisfaction among the students who performed the VR experience, while the Desktop group reported less satisfaction although still far better than the control (Webcam) group. Although the higher satisfaction with the VR experience might come from the novelty effect of the VR environments, this effect hardly appears to be the only reason, because the Desktop game also achieved high satisfaction levels, which may partially be due to the hands-on learning strategy of the serious game compared with conventional learning methodologies, as pointed out in previous works (Makransky et al. 2019). Besides, this difference between the VR and the Desktop groups could be linked to the different interfaces, showing the VR controllers and the higher feeling of natural manipulation of the physical configuration in comparison with keyboard and mouse controls, as both objective and subjective indicators show. This effect has previously been reported in some general studies: usability plays a major role in student satisfaction reports (Chen et al. 2013) and some with similar learning tasks (Zhou et al. 2018), as is also proposed in this research. It was observed that the VR participants spent more time on the Adaptation level, but they were faster than the Desktop group in the Assignment level. This first result on higher satisfaction with VR was expected; it is a common conclusion in VR studies compared with conventional teaching methodologies (Checa and Bustillo 2020a): young students perceive VR within education as an exciting and challenging opportunity once a minimum of expertise in the VR interface is gained. Gaining greater expertise is highly recommended to provide VR users with more time to explore and to adapt to the learning environment, in order to minimize the "novelty effect” of this technology.

In the bibliography, the higher satisfaction with VR methodologies is commonly connected with higher learning rates, although these conclusions are, in most cases, never statistically evaluated with a proper sample of users (Checa and Bustillo 2020a). The analysis of the tests on academic performance showed a significant difference in the learning rates for the serious games users in opposition to the control (Webcam) group, for both the VR and the desktop game versions. This improvement suggests that serious games are a suitable tool for enhancing learning. Likewise, the VR game showed better results than the desktop game, which suggests that the increased learning in the VR condition was not a direct result of the game, as it was the same in both cases. Instead, the learning appears to be attributable to both 3D immersion and the interactivity of the VR environment, as recent research has outlined (Buttussi and Chittaro 2017).

**Fig. 10:**
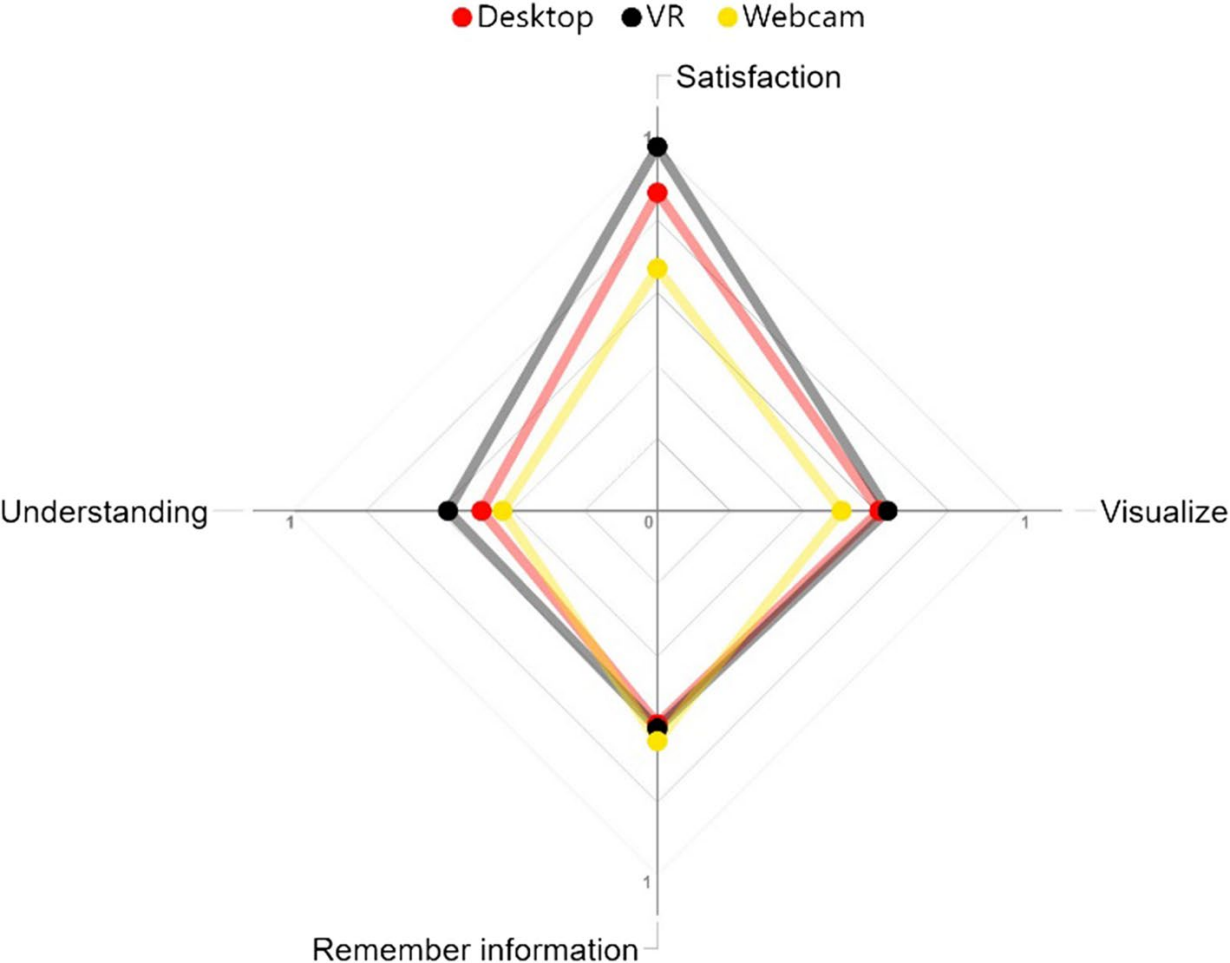
Effect of the three teaching methodologies in the different learning outcomes

However, not all kinds of knowledge are especially suitable for VR games. Figure 10 illustrates the results obtained with the different satisfaction surveys and the evaluation tests of the educational experience, showing the averaged mark (being 1.0 the maximum satisfaction/acquisition rate) of each group for the four considered outputs. The results revealed different learning stages as defined in Bloom's taxonomy: for example, the slightly worse results for information recall from the serious games groups in comparison with the traditional learning group. This aspect has been mentioned in the literature (Checa and Bustillo 2020b) and is based on the cognitive theory of multimedia learning, which predicts that students will learn more with a well-designed slide presentation, even though they may report lower levels of interest and motivation (Parong and Mayer 2018). Although there were very poor improvements at recalling information with VR, the final analysis of the most complex questions for this kind of knowledge shown in Sect. 5 showed a clear improvement for those questions from the pre to the post-questionnaire. The conclusion was that all the proposed teaching methodologies helped student learning processes.

On the other hand, VR and conventional conditions were more conducive to ‘understanding’ than the Desktop, as some previous results have also pointed out (Allcoat and von Mühlenen 2018). Compared to observation-based learning methodologies, the chance to interact with components can significantly enhance the acquisition of different types of knowledge (Borsci et al. 2016) among users and their performance of well-established procedures (Buttussi and Chittaro 2017). Finally, visual recognition and knowledge is clearly better acquired in both interactive and immersive VR environments compared with traditional approaches, showing slightly better results than the Desktop group. This result was especially significant, because the proposed questions evaluated each user’s ability to extrapolate the acquired knowledge to new scenarios (e.g., identifying computer components within a significantly different computer cage than the one displayed in the serious game). This type of evaluation is significantly different from the one used in VR training games where it is mainly the same items and procedures that usually appear in the tests (Abich et al. 2021). It appears clear that VR is of great potential for the acquisition of this kind of knowledge, as outlined in some previous studies (Checa and Bustillo 2020b; Molina-Carmona et al. 2018).

## Conclusions

Firstly, a VR serious game for teaching computer hardware assembly to undergraduate students in an introductory study unit to Computer Science has been presented in this research. The game has been designed to mitigate various obstacles detected in VR educational applications such as the "novelty effect" and user’s astonishment due to the technological novelty or lack of interface control. Tutorial stages and natural game interfaces have been used for this purpose. The game is designed as a hands-on learning environment to increase interest, because students prefer practice-oriented learning content rather than memorization of facts. This design follows the idea that rather than better teaching of traditional knowledge, the real potential of VR is found in "learning by doing", which is usually very difficult to apply in traditional classes.

Secondly, the VR-serious game has been integrated in a complete learning experience and compared with another two learning methodologies. Those reference methodologies were based (1) on the same serious game, but for a desktop PC, and (2) a conventional lecture adapted to online learning times, where the computer hardware is presented by the teacher with a webcam and an open computer, although the student is watching and cannot manipulate any components. An extensive group of students (*n* = 77) was selected with a major proportion (*n* = 40) in the VR group, to evaluate the performance of these 3 learning methodologies. The analysis of a knowledge pre-test, a satisfaction/usability test, a knowledge post-test, and some performance indicators have yielded the following conclusions:

- Student satisfaction: the game in both its desktop and VR versions significantly improved student satisfaction compared with the traditional teaching method. Besides, students gave more positive learning beliefs ratings to VR (*p* = 0.016 < 0.05) than to the desktop game or conventional class methods. This result is especially interesting against the backdrop of the COVID-19 crisis and student confinement, when student health and well-being should receive special support: if we enjoy learning, obstacles might appear smaller.
- Game usability: the students thought it was significantly easier to interact in VR than the desktop PC version controlled by a keyboard and a mouse. Besides, they found that the VR environment was slightly easier to control than those playing the game on a desktop.
- Information recall: the slightly worse results of the serious games groups at recalling information than the traditional learning group may be highlighted.
- Understanding: on average, VR showed a slightly better performance. However, VR students performed significantly better that the desktop and the Webcam students in response to questions on the time that is required for RAM assembly and improved their results in comparison with the pre-test. This leads us to suggest that the sensation of immersion was better than the other two options at helping students to extract applied knowledge for real life.
- Visual recognition: students who used the VR application showed significantly better visual recognition than the group that received the traditional class and had slightly better results than the students using the desktop serious game.
- Performance: the VR group performed the exercise faster and made fewer errors than the students playing the same game on the desktop. A relationship can be established between the greater usability that users perceive of VR with the time required to complete the learning task. In addition, the fact that they spent more time at the introductory level may have meant that the VR users were more focused on the subsequent levels that are relevant for learning.

All these conclusions point to the following: (1) the strong potential of VR serious games to improve students well-being in times of isolation due to higher learning satisfaction; (2) the positive effect of learning theoretical knowledge, but specially for developing understanding and connection between different concepts; (3) although computer hardware might not be a topic that is closely connected with spatial knowledge, as with many topics such as Cultural Heritage, Mechanical Engineering and Architecture, VR still provides significant advantages compared to other methodologies for student absorption of visual knowledge; and (4) the usability of the game and the use of tutorials are directly connected with user satisfaction and game performance.

This research has helped to answer some questions, but many others still remain open for future research. The effect of VR applications on a continuous and stable learning process should be reported as other authors have stated (Ray and Deb 2016), because once the students feel comfortable and competent with the VR interfaces and the game design, then the learning outcomes may be boosted. For instance, the impact of the novelty effect should be quantified. Besides, not only longer experiences, but also novel learning methodologies have to be developed to assure the right overlap between conventional lectures and autonomous RV learning sessions. For instance, home VR solutions should be tested and compared with classroom high-end VR solutions as the one presented in this research. Finally, new learning topics where a higher degree of visualization and experiential awareness is required must be tested, to establish the limits of VR in relation to learning tasks. For instance, VR solutions for dangerous-task training, like electrical hazards in industrial equipment maintenance.

## Supplementary Information

Below is the link to the electronic supplementary material.

Supplementary file 1 (DOC 653 kb)

Supplementary file 2 (DOC 89 kb)

Supplementary file 3 (DOC 4958 kb)

Supplementary file 4 (XLSX 58 kb)
