# Supplementary material for: Immersive virtual-reality computer-assembly serious game to enhance autonomous learning
Source: Virtual Real. 2021 Dec 23:1–18. Online ahead of print. doi: 10.1007/s10055-021-00607-1 (PMC8695959; doi:10.1007/s10055-021-00607-1)
Supplement: Supplementary file 1 — (DOC 653 kb) [file 10055_2021_607_MOESM1_ESM.doc]

Name ___________________________________________ Gender __________________

Age ____ Studies (for those who access this degree) _______________________________

**Have you had any experience in Virtual Reality before?:** Yes □ No □

If yes, with what type of device*?* □ Mobile Device (Cardboard type)

□ Virtual reality head mounted display (type Oculus rift, Htc Vive...) □ I don't know what device it was

**What kind of experience in Virtual Reality: (Educational, game...)_________________ ___________________________________________________________________________**

**Have you ever seen a computer inside? Describe the situation.** E.g. (Studying a FP module... I have assembled my own PC or replaced parts...___________________________________________________

**__________________________________________________________________________**

**__________________________________________________________________________**

**How long do you think it takes to mount two RAM modules and the graphics card in a computer? (in minutes) _______________________________________________**

**Mark with an X the box you consider correct**

**1. Do you think you would be able to increase the RAM memory of your computer?**

Yes □

No □

**2. What name are the physical parts of a PC identified by?**

□ Software

□ Hardware

□ Freeware

□ Peripherals

**3. What is the main function of the motherboard?**

□ Processing the instructions given to the computer

□ Linking all computer functions

□ Allow the computer to display videos

□ Acting as the computer's brain

**4. The computation speed of a processor is measured in**

□ Watios

□ Voltios

□ GHz

□ MB

**5. The RAM memory:**

□ Stores information on the computer permanently

□ Controls the flow of computer data

□ Transforms digital data into an analog signal

□ Stores the data being used at the present time

**6. Which of these storage devices can store the most data?**

□ Hard Disk □ RAM memory

□ ROM memory □ DVD-R

**7. What are the main manufacturers of Video Card GPUs?**

□ Nvidia and Gigabyte.

□ Nvidia and AMD

□ MSI and AMD

□ Asus and Nvidia.

**8. The electronic component that serves to supply electricity to the computer is:**

□ Socket

□ Power supply

□ Control unit

□ Chipset

**9. A hard disk of type HDD with respect to an SSD is:**

□ Larger

□ Smaller

□ They are the same size

**10. Complete the number of each component according to the image above.**

***Motherboard ____ CPU and CPU cooler _____ RAM memory _____***

***Memory _____***
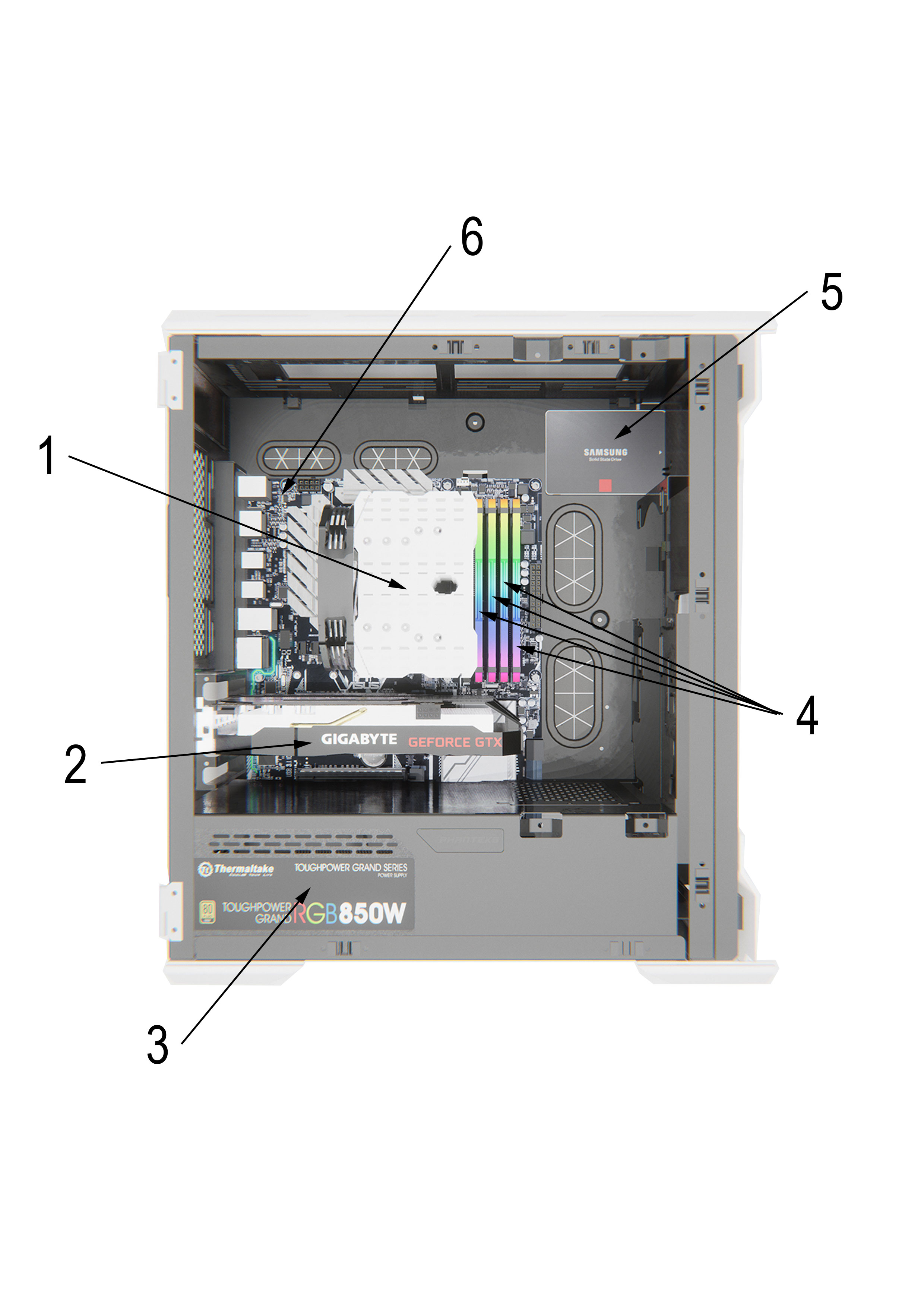
 ***Graphics card _____ Power Supply ______***
