# Supplementary material for: Immersive virtual-reality computer-assembly serious game to enhance autonomous learning
Source: Virtual Real. 2021 Dec 23:1–18. Online ahead of print. doi: 10.1007/s10055-021-00607-1 (PMC8695959; doi:10.1007/s10055-021-00607-1)
Supplement: Supplementary file 2 — (DOC 89 kb) [file 10055_2021_607_MOESM2_ESM.doc]

Name ___________________________________________ Time spent ______

**Have you had any experience in Virtual Reality before?** Yes □ No □

**Have you suffered dizziness from the experience?**

□ Severe, I had to leave the experience □ Moderated □ Mild □ Non-existent

**Being 1 Nothing, 2 Little, 3 Quite a lot, 4 A lot and 5 Very much, please answer the following questions:**

**Have you enjoyed the experience with the game?**

□1 □2 □3 □ 4 □5

**Do you think this educational game will be useful for your learning?**

□1 □2 □3 □ 4 □5

**Have you been able to control the game without problems?**

□1 □2 □3 □ 4 □5

**At each step, did you know what to do?**

□1 □2 □3 □ 4 □5

**Is the information provided within the game clear?**

□1 □2 □3 □ 4 □5

**Did the interaction with the virtual environment seem natural?**

□1 □2 □3 □ 4 □5

**I found the visual aspects of the virtual environment to be realistic**

□1 □2 □3 □ 4 □5

**I thought the interaction devices (Oculus Touch) were easy to use.**

□1 □2 □3 □ 4 □5

**The devices (Oculus Touch) that controlled the interactions in the game seemed natural.**

□1 □2 □3 □ 4 □5

**I have felt confident holding objects in the virtual environment.**

□1 □2 □3 □ 4 □5

**I have felt confident to move and interact with the virtual environment at the end of the experience.**

□1 □2 □3 □ 4 □5

**Remember what the sign on the wall said?**

___________________________________________________________________________

Continued on the back of this page...

**Do you remember what was on the table behind you?**

__________________________________________________________________________

**In your opinion, what are the positive points of the experience?**

___________________________________________________________________________

___________________________________________________________________________

___________________________________________________________________________

___________________________________________________________________________

**In your opinion, what are the negative points of the experience?**

___________________________________________________________________________

___________________________________________________________________________

___________________________________________________________________________

___________________________________________________________________________

**Any suggestions for improving this class?**

___________________________________________________________________________

___________________________________________________________________________

___________________________________________________________________________

___________________________________________________________________________

Name ___________________________________________

**Being 1 Nothing, 2 Little, 3 Quite a lot, 4 A lot and 5 Very much, please answer the following questions:**

**The experience with the class of assembly of a computer through the screen has been interesting to me**

□1 □2 □3 □ 4 □5

**Do you think this teaching system will be useful for your learning?**

□1 □2 □3 □ 4 □5

**Have you been able to observe the computer parts well at all times?**

□1 □2 □3 □ 4 □5

**At every step, did you understand what you were seeing?**

□1 □2 □3 □ 4 □5

**Is the information provided within the class clear?**

□1 □2 □3 □ 4 □5

**In your opinion, what are the positive points of the experience?**

___________________________________________________________________________

___________________________________________________________________________

___________________________________________________________________________

___________________________________________________________________________

**In your opinion, what are the negative points of the experience?**

________________________________________________________________________

___________________________________________________________________________

___________________________________________________________________________

___________________________________________________________________________

**Any suggestions for improving this class?**

___________________________________________________________________________

___________________________________________________________________________

___________________________________________________________________________

___________________________________________________________________________

Name ___________________________________________ Time spent ______

**Being 1 Nothing, 2 Little, 3 Quite a lot, 4 A lot and 5 Very much, please answer the following questions:**

**Have you enjoyed the experience with the game?**

□1 □2 □3 □ 4 □5

**Do you think this educational game will be useful for your learning?**

□1 □2 □3 □ 4 □5

**Have you been able to control the game without problems?**

□1 □2 □3 □ 4 □5

**At each step, did you know what to do?**

□1 □2 □3 □ 4 □5

**Is the information provided within the game clear?**

□1 □2 □3 □ 4 □5

**Did the interaction with the virtual environment seem natural?**

□1 □2 □3 □ 4 □5

**I found the visual aspects of the virtual environment to be realistic**

□1 □2 □3 □ 4 □5

**I thought the interaction devices (mouse and keyboard) were easy to use.**

□1 □2 □3 □ 4 □5

**The devices (mouse and keyboard) that controlled the interactions in the game seemed natural.**

□1 □2 □3 □ 4 □5

**I have felt comfortable selecting objects in the virtual environment.**

□1 □2 □3 □ 4 □5

**I have felt competent to move and interact with the virtual environment at the end of the experience.**

□1 □2 □3 □ 4 □5

**Do you remember what the sign on the wall said?**

___________________________________________________________________________

**Do you remember what was on the back table?**

__________________________________________________________________________

Continued on the back of this page...

**In your opinion, what are the positive points of the experience?**

___________________________________________________________________________

___________________________________________________________________________

___________________________________________________________________________

___________________________________________________________________________

**In your opinion, what are the negative points of the experience?**

___________________________________________________________________________

___________________________________________________________________________

___________________________________________________________________________

___________________________________________________________________________

**Any suggestions to improve this video game?**

___________________________________________________________________________

___________________________________________________________________________

___________________________________________________________________________

___________________________________________________________________________
