# Supplementary material for: Immersive virtual-reality computer-assembly serious game to enhance autonomous learning
Source: Virtual Real. 2021 Dec 23:1–18. Online ahead of print. doi: 10.1007/s10055-021-00607-1 (PMC8695959; doi:10.1007/s10055-021-00607-1)
Supplement: Supplementary file 3 — (DOC 4958 kb) [file 10055_2021_607_MOESM3_ESM.doc]

Name and Surname __________________________________________________________

**Mark with an X the box you consider correct**

**1. The motherboard:**

□ Coordinates the operation of all devices connected to the computer

□ Connects the different components of an equipment

□ Stores program data temporarily

□ It is the brain of the computer

**2. Which of the following statements is correct knowing that the Socket is the connection place of the processor on the motherboard:**

□ All motherboards have the same type of socket

□ Not all motherboards have the same type of socket

□ There are different types of sockets, but they are all compatible with each other

□ Each brand has a unique type of socket shared by all its CPUs

**3. Where do we connect the RAM?**

□ On the motherboard socket.

□ In the motherboard memory sockets.

□ On the mainboard IDE connector.

□ In the PCI slot on the motherboard.

**4. An SSD type memory:**

□ No vibration due to the absence of moving parts

□ The rotation of your discs may cause slight vibrations

□ It has mechanical parts that can be damaged by rough movements

□ Magnetism can remove your data

**5. The graphic card:**

□ It has integrated video outputs and is accessible from outside the PC case

□ It has integrated video outputs and is accessible from inside the PC case

□ It does not have integrated video outputs and the connection is made on the motherboard

□ It only processes graphics and has no video outputs

**6. A power supply with insufficient voltage for our components:**

□ The computer will not turn on

□ Can cause system crashes

□ It will be more efficient by consuming less energy

□ The voltage is indifferent for the components what matters is the efficiency

**7. Which element is not anchored to the base plate:**

□ RAM

□ CPU

□ Hard disk

□ Graphics card

**8. We place the CPU on the motherboard...**

□ Before placing the CPU cooler

□ After placing the CPU cooler

□ The CPU is not placed on the motherboard

**9. The RAM memory:**

□ Stores information on the computer permanently

□ Controls the flow of computer data

□ Transforms digital data into an analog signal

□ Stores the data being used at the present time

**10. An SSD type hard disk:**

□ It has less capacity and is smaller than an HDD

□ It has more capacity and is larger than an HDD

□ It has more capacity and is smaller than an HDD

□ It has less capacity and is larger than an HDD

**11. The graphics card on the motherboard:**

□ It is placed horizontally

□ It is placed vertically

□ It is placed at the top

□ Not placed on the motherboard

**12. The power supply:**

□ In modern boxes we place it on top to improve the air flow

□ In modern boxes we place it in the lower part to improve the air flow

□ In modern boxes it is placed outside the box so as not to transfer the heat to the other components

□ It is so small that it does not affect the air flow and barely produces any heat

**13. The most powerful CPUs:**

□ They do not heat up when soldered to the base plate

□ Powerful CPU coolers are integrated into the microchip to dissipate the heat

□ Need better CPU coolers to ensure stable operation

□ They do not need CPU coolers as their components evacuate heat better

**14. A computer BIOS is usually stored:**

□ In a ROM memory

□ In a RAM memory

□ In the CPU cache

□ Nowhere, since the BIOS is not currently used

**15. What is the main function of the motherboard?**

□ Processing the instructions given to the computer

□ Linking all computer functions

□ Allow the computer to display videos

□ Functioning as the computer's brain

**16. Would you dare to try to increase your computer's RAM memory after this experience?**

Yes □ No □

**17. How long do you think it takes to mount two RAM modules and the graphics card in a computer? (in minutes) _________________________________________**

**18. Why do you think there is so much free size inside the computer? _________________________________________________________________________________________________________________________________________________________________________________________________________________________________**

**19. What element of the computer do you think will most restrict the final capabilities of the computer and why? _________________________________________________________________________________________________________________________________________________________________________________________________________________________________**


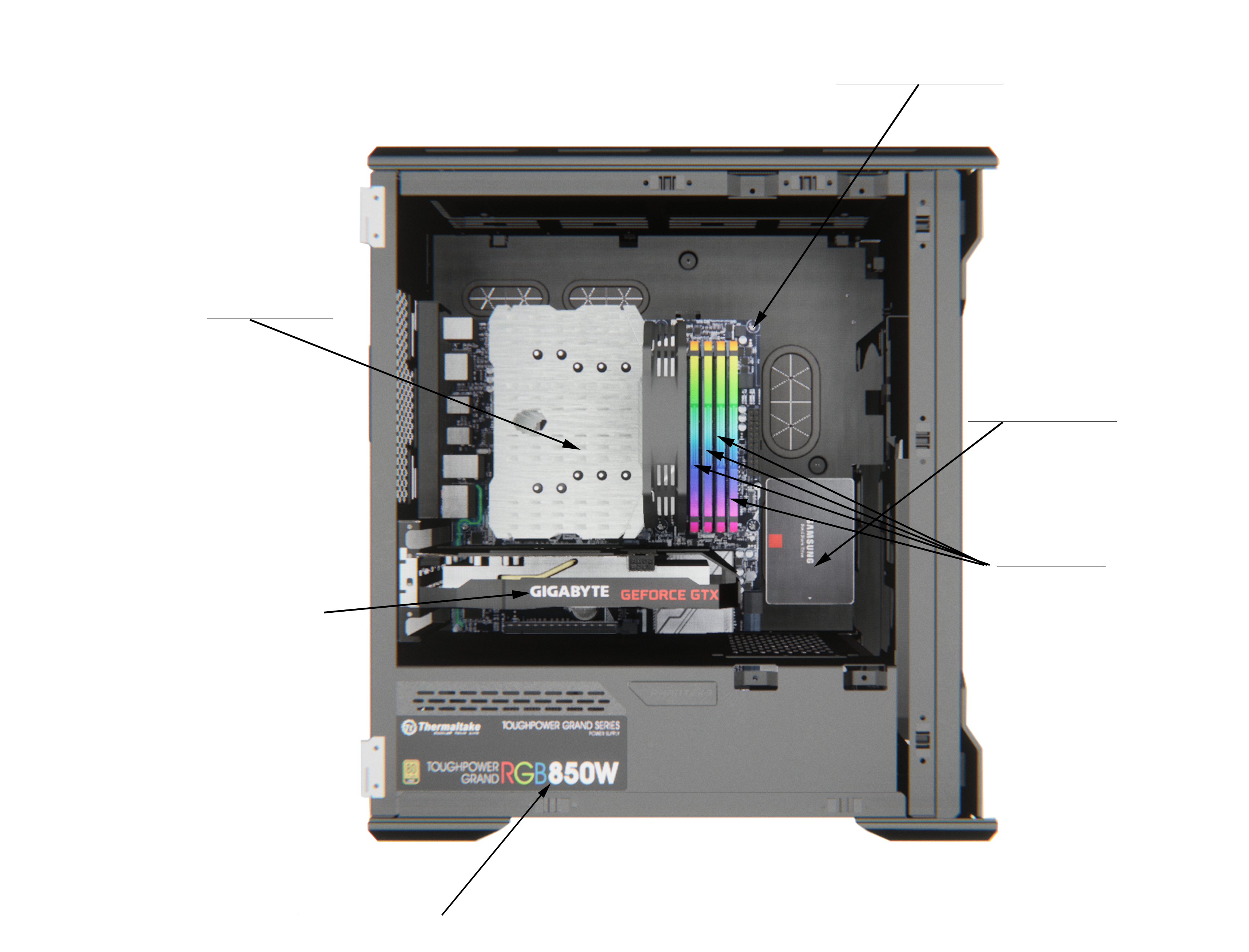
**20. Write the name of each component in the space**

Continue on the back of this page...

**21. Write the name of each component in the space**

**
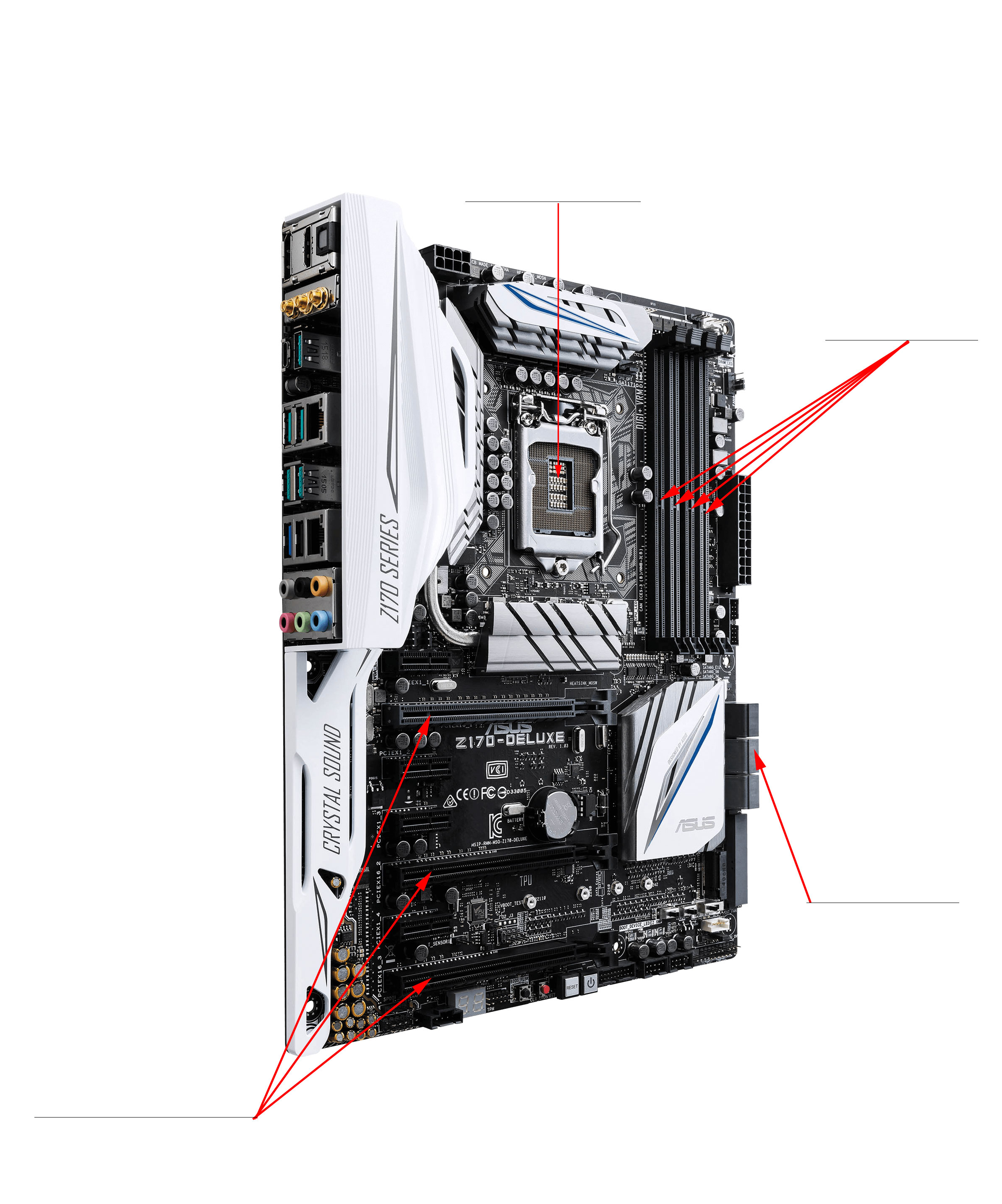
**
